# The complete mitochondrial genome sequence of *Centropus bengalensis* (Lesser Coucal)

**DOI:** 10.1080/23802359.2020.1731349

**Published:** 2020-02-28

**Authors:** Ya-Lin Huang, Xiao-Qi Sun, Jing Jiang, Yi-Ling Fei, Sen-Lin Hou

**Affiliations:** a Key Laboratory of State Forest and Grassland Administration on Wildlife Evidence Technology, Nanjing Forest-Police College, Nanjing, China;; b Criminal Science and Technology Faculty of Nanjing Forest Police College, Nanjing, China

**Keywords:** *Centropus bengalensis*, Lesser Coucal, mitogenome

## Abstract

We first reported the mitochondrial genome of *Centropus bengalensis*. The mitogenome of *C. bengalensis* contains 17,117 base pairs. The overall base composition of complete mitogenome is 28.15% A, 27.95% T, 21.86% C, and 22.04% G, with 43.90% of the GC content. All genes exhibit the typical mitochondrial gene arrangement and transcribing directions. Phylogenetic analysis of 4 Centropus species was performed based on the sequence of cytochrome b gene using the neighbor-joining method and the Kimura 2-parameter model in MEGA 7.0.

*Centropus bengalensis* (Lesser Coucal) majorly distributes in the south of the Yangtze River in China. Meanwhile, it is widely found in South, East, and Southeast Asia. Because the local people are used to killing *C. bengalensis* as raw material of chicken wine, the number of wild population of the bird has decreased dramatically, and it has been listed as an endangered bird since 1989 by the Chinese government. Compared with other protected species, *C. bengalensis* received less attention. Hence, less information on the complete mitogenome of *C. bengalensis* was available. In this study, we characterized the complete mitogenome of *C. bengalensis* by using next-generation sequencing techniques.

Tissue samples of *C. bengalensis* were collected from Lai An County (118°43′N, 32°45′E) in Chuzhou City, Anhui Province, in September 2019 and after sampling, the specimens (NJFPC-2019459) were stored in the animal specimens museum of Nanjing Forest-police College.

mtDNA was isolated (Chen et al. 2011), 1 μg of purified mtDNA was fragmented and used to construct short-insert libraries (insert size 430 bp) according to the manufacturer’s instructions (Illumina), and then sequenced on the Illumina Hiseq 4000 (Borgstrom et al. 2011). Prior to assembly, raw reads were filtered firstly. Then, the filtered reads were assembled into contigs using SOAPdenovo2.04 (Luo et al. 2012). The mitochondria genes were annotated using homology alignments and denovo prediction, and the EVidenceModeler v1.1.1 (Haas et al. 2008) was used to integrate gene set. A whole mitochondria genome Blast (Altschul et al. 1990) search was performed against five databases.

The length of complete mitogenome of *C. bengalensis* is 17,117 bp (GenBank accession: MN996304). The complete mitogenome is relatively AT rich (that is 56.10% vs 43.90% of the GC content) with the following nucleotide compositions: 28.15% A, 27.95% T, 21.86% C, and 22.04% G. The mitogenome consists of 22 transfer RNA genes, 13 protein-coding genes, two ribosomal RNA genes (i.e., one rrnL and one rrnS), and one control regions. Most of the genes are encoded on the heavy (H) strand, except for those encoding nad6 and eight tRNAs. All genes follow the typical mitochondrial gene arrangement and transcribing directions, which is identical to most vertebrates (Huang et al. 2019; Lu et al. 2019).

Phylogenetic analysis of 4 Centropus species was performed based on the complete mitogenome of *C. bengalensis* and the nucleotide sequences of cytochrome b (Cyt b) of other 3 Centropus species, using the neighbour-joining method and the Kimura 2-parameter model in MEGA 7.0, with 1000 bootstrap replicates (Kumar et al. 2016). Phylogenetic tree showed that the *C. bengalensis* is closely related to *Centropus phasianinus* (Figure 1). The genome information obtained here could contribute to future studies on molecular evolution and wildlife protection in *C. bengalensis*.

**Figure 1.**
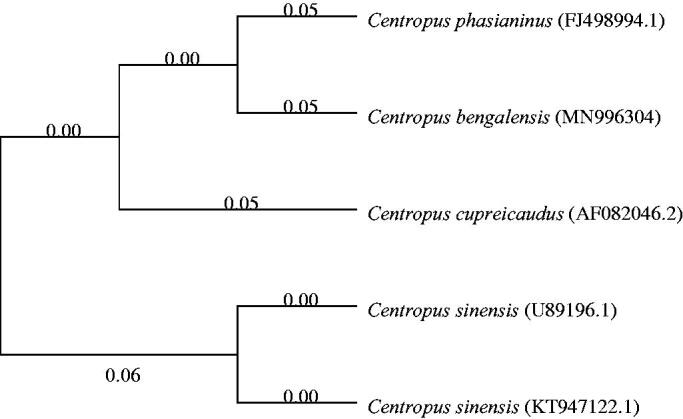
Phylogeny of 4 Centropus species based on the complete mitogenome of *C. bengalensis* and the nucleotide sequences of cytochrome b of other 3 Centropus species using neighbour-joining method.
